# A Review of Epigenetics of PTSD in Comorbid Psychiatric Conditions

**DOI:** 10.3390/genes10020140

**Published:** 2019-02-13

**Authors:** Caren J. Blacker, Mark A. Frye, Eva Morava, Tamas Kozicz, Marin Veldic

**Affiliations:** 1 Department of Psychiatry and Psychology, Mayo Clinic, Rochester, MN 55905, USA; Blacker.Caren@mayo.edu (C.J.B.); MFrye@mayo.edu (M.A.F.); 2 Center for Individualized Medicine, Mayo Clinic, Rochester, MN 55905, USA; Morava-Kozicz.Eva@mayo.edu (E.M.); Kozicz.Tamas@mayo.edu (T.K.); 3 Clinical Genomics, Mayo Clinic, Rochester, MN 55905, USA

**Keywords:** epigenetic, post-traumatic stress disorder, psychiatric disorders

## Abstract

Post-traumatic stress disorder (PTSD) is an acquired psychiatric disorder with functionally impairing physiological and psychological symptoms following a traumatic exposure. Genetic, epigenetic, and environmental factors act together to determine both an individual’s susceptibility to PTSD and its clinical phenotype. In this literature review, we briefly review the candidate genes that have been implicated in the development and severity of the PTSD phenotype. We discuss the importance of the epigenetic regulation of these candidate genes. We review the general epigenetic mechanisms that are currently understood, with examples of each in the PTSD phenotype. Our focus then turns to studies that have examined PTSD in the context of comorbid psychiatric disorders or associated social and behavioral stressors. We examine the epigenetic variation in cases or models of PTSD with comorbid depressive disorders, anxiety disorders, psychotic disorders, and substance use disorders. We reviewed the literature that has explored epigenetic regulation in PTSD in adverse childhood experiences and suicide phenotypes. Finally, we review some of the information available from studies of the transgenerational transmission of epigenetic variation in maternal cases of PTSD. We discuss areas pertinent for future study to further elucidate the complex interactions between epigenetic modifications and this complex psychiatric disorder.

## 1. Introduction

Post-traumatic stress disorder (PTSD) is a complex psychiatric condition. It is acquired following exposure to a stressor that the individual perceives as threatening to the physical and/or psychological integrity of self. Although the majority of individuals in the general population exposed to such a stressor develop no long-term sequelae, fewer than 10% will develop PTSD [1,2]. In the United States, PTSD is typically diagnosed using clinical criteria from the Diagnostic and Statistical Manual of Mental Disorders 5 (DSM-5) [3]. These criteria include exposure to a traumatic stressor with subsequent intrusion symptoms (e.g., flashbacks, nightmares, physiological reactivity), avoidance behaviors, negative mood/thoughts, and/or alterations in arousal (e.g., hypervigilance, exaggerated startle, sleep disturbance). The lifetime prevalence of PTSD among American adults is 6.8% (men 3.6% and women 9.7%) [1,2]. Suicide rates are elevated in PTSD patients in most studies; although the statistical relationships are complex, especially since comorbid psychiatric diagnoses are frequently present. Thus, PTSD is associated with a heavy personal, clinical, and economic burden of disease. Many factors affect the development and phenotype of PTSD: genetic vulnerability; the type, frequency, intensity, and recurrence of trauma; the age and developmental stage at which trauma occurs; and comorbid psychiatric and substance use disorders. There are also speculated to be epigenetic factors involved in susceptibility to PTSD, the severity of symptoms, and the clinical course; these factors are still being elucidated and are described in this article. A longstanding clinical conundrum has been why most individuals are resilient to trauma, while only a minority develops PTSD. Another question has been the degree to which PTSD is causal for comorbid psychopathologies, and vice versa [4]. 

Epigenetics is the study of changes in gene expression that do not involve changes to the genetic sequence. Epigenetic mechanisms include DNA methylation, histone modification, and RNA-associated silencing. DNA methylation, mediated by DNA methyltransferases, typically decreases or silences gene transcription and in mammals occurs most frequently at the cytosine of a CpG site (a DNA sequence where cytosine is 5-prime to guanine, separated by a phosphate). DNA methylation can also occur at non-CpG sites, including adenine bases, particularly CpApC sequences [5]. Differentially methylated regions (DMRs) are genomic regions with different degrees of methylation in different samples (across cells, tissues, or subjects). 

Histones can be modified by the addition of one or more chemical groups (e.g., acetyl, methyl, ubiquitin, phosphorus, small-ubiquitin-like modifier, citrulline, and ribose). Histone acetylation is undertaken by histone acetyl transferases while deacetylation is performed by histone deacetylases (HDACs). The effect of histone modifications on transcription is highly variable: histone acetylation opens chromatin conformation to promote transcription; histone phosphorylation is often a marker of DNA damage, while the translational effect of mono-, di-, or tri-methylation depends on which histone protein is modified. 

RNA-associated silencing is a mechanism by which non-coding RNA (nc-RNA) downregulates gene expression. Long nc-RNAs (lncRNA) are transcripts greater than 200 nucleotides; short nc-RNAs come in a huge variety of molecules including micro-RNA (miRNA), small interfering RNA (siRNA), and piwi-interacting RNA (piRNA).

The development of PTSD involves multiple neural networks, especially those for emotion, memory, and learning. Animal studies have modeled PTSD using fear conditioning, i.e., associating an aversive stimulus (the trauma) with a neutral context or stimulus. Certain paradigms add extinction learning, so that the animal stops anxiety behavior in the context of the neutral stimulus once it is no longer paired with the aversive stimulus. Epigenetic mechanisms have been uncovered in such paradigms, with HDACs increasing memory formation in fear extinction [6]. Fear conditioning lacks the complexity of the clinical syndrome of PTSD in humans, so prolonged, variable-stress models continue to be explored [7]. Previous reviews have explored how PTSD can affect the subsequent development of psychopathology [8] or genetic and epigenetic associations with PTSD [9,10]. In this review, we discuss epigenetic changes in PTSD in the context of other psychiatric comorbidities, potential environmental co-contributors, and recommendations for future studies of these important clinical questions.

## 2. Candidate Genes in PTSD

Genetic and epigenetic influences work in tandem, though a genetic variant related to PTSD does not automatically infer an epigenetic association. However, both genetic variants and epigenetic regulation can alter gene expression and thereby influence clinical phenotype, albeit by different mechanisms. Thus, understanding target genes; their protein functions; and the relationship between significant single nucleotide polymorphisms (SNPs), CpG sites, and CpG islands (regions with a high frequency of CpG sites) is essential to the understanding of epigenetics. Multiple studies have uncovered genetic contributions to PTSD risk and symptomatology. Candidate genes have encoded proteins as varied as neurotransmitter and steroid receptors and transporters [11,12,13,14], transcription factors [11,15], regulators of cell division and growth [16,17], immunoregulatory signals [18], and cell adhesion molecules [16]. However, few studies have been conducted to examine or replicate genetic associations in PTSD [17,19,20,21]. This is especially problematic as many of those studies have examined small populations of PTSD patients, and genetic polymorphisms frequently have only very small effect sizes on phenotype and disease risk. 

As an area of research still in its infancy, no gene has been unequivocally linked with PTSD. It is important that this knowledge gap is rectified to continue to understand genotypic contributions to this disease as well as to interpret epigenetic signatures. In order to provide a reference for future research, we used Malacards: The Human Disease Database (https://www.malacards.org) to collect studies reporting protein-coding genes associated with human cases of PTSD. We also gleaned further protein-coding gene candidates from the citations of these studies and have collated them in Table 1. This table provides a reference point for researchers. These studies have huge variations in power and sample size, and the population differences (race, sex, age, trauma exposure, and trauma type) are also significant. The study designs and resulting reported mechanisms of association also differ. Outside the context of a formal PTSD diagnosis, many other protein-coding and non-protein-coding genes have been investigated for their contribution to neuroanatomical, behavioral, and clinical features associated with PTSD, and we refer interested readers to these studies [22,23,24].

## 3. Overview of Epigenetic Markers in PTSD

### 3.1. Methylomics

As with genetic associations, there are still very few epigenetic studies of PTSD cohorts. For example, we are only aware of four genome-wide methylation studies in PTSD [17,19,20,21], making this a rich field for future research. Additionally, these studies use peripheral tissue for DNA methylation analysis, and brain tissue has not yet been available for confirmation that the findings are reflected in the central nervous system. These genome-wide methylomic studies in PTSD have reported varying results. A genome-wide gene expression study of 12 PTSD subjects and 12 trauma-exposed without-PTSD controls found 3989 genes significantly upregulated in PTSD and three downregulated (*p* < 0.05 adjusted for multiple comparisons; fold change >2) but no significant (*p* < 0.05) differences in DNA methylation [19]. This same study reported that olfactory function and immune system gene expression were upregulated. However, other studies have found the downregulation of immune system genes. A genome-wide DNA methylation study of Australian Vietnam combat veterans reported the *DOCK2* (Dedicator of cytokinesis 2) gene was significantly downregulated and associated with decreased methylation in PTSD [17]. The DOCK2 protein is expressed solely in leukocytes and appears to have a significant role in the chemotaxis of immune cells. A compromised immune response was suggested both clinically and epigenetically in adult PTSD subjects obtained from a population sample of a large, industrial city in the United States (Detroit, MI) [21]. Compared to healthy controls, PTSD subjects had uniquely unmethylated genes related to immune and inflammatory response, and cytomegalovirus antibodies were significantly higher in PTSD subjects, a possible marker of compromised immune systems. Of these differentially methylated genes, only one (*MAN2C1*) was later demonstrated to modify the risk of PTSD in the context of cumulative trauma [38]. *MAN2C1* encodes a mannosidase involved in the degradation of glycoprotein-derived sugars and regulating apoptosis [39]. α-Mannosidase-like activity is also found in a similar class of proteins that remove misfolded polypeptides within cells [40]. Since the accumulation of misfolded proteins and alterations in apoptosis have been implicated in PTSD pathology [41], *MAN2C1* would be an interesting candidate gene for further study. 

African-American patients from a large, urban area were assessed for PTSD and stressful life events [42]. There was no change in global DNA methylation levels in subjects with either a history of childhood abuse or increased total life stress, but global DNA methylation increased in PTSD subjects. A significant differential DNA methylation was observed in PTSD subjects at CpG sites in 5 genes: decreased in *TPR* (involved in trafficking across the nuclear membrane) and *ANXA2* (calcium-regulated membrane-binding protein involved in signal transduction and cellular growth) and increased in *CLEC9A* (activation receptor on myeloid cells), *ACP5* (glycoprotein with elevated expression in leukemias), and *TLR8* (pathogen recognition and innate immunity activation). The increased methylation of CpG sites in the genes *CXCL1* and *BDNF* were associated with PTSD and comorbid total life stress.

The same, predominantly African-American, population was then used to examine differential methylation in women. This study found a significantly higher methylation of the CpG site cg22937172 in histone deacetylase 4 gene (*HDAC4*) in PTSD cases compared to healthy controls [20]. Interestingly, cg22937172 methylation was also associated with lower blood estradiol. The same study also assessed mouse amygdala *HDAC4* levels and found that fear conditioning (as an animal PTSD model) was associated with higher *HDAC4* expression and that this was modified by estrogen levels. Replication of a direct estrogen effect in humans would be difficult in naturalistic studies but, if possible, might provide insight into the sex differences of PTSD rates as well as a potential epigenetic mechanism. 

### 3.2. Histone Modifications

Studies of histone modifications in rodent PTSD models have found brain region and environmental differences. Adult male rats underwent fear conditioning and brain histone acetylation measurement [43]. Histone H3 lysine 9 (H3K9ac) and Histone H4 lysine 5 acetylation (H4K5ac) both increased significantly in lateral/basal/centrolateral amygdala after fear conditioning. H3K9ac and H4K5ac also increased in centromedial amygdala and prelimbic-prefrontal cortex (PL-PFC) but only after fear learning. There was differential H4K5ac in prefrontal cortex, significantly decreasing in infralimbic-prefrontal cortex (IL-PFC) and increasing in PL-PFC after fear learning. Histone acetylation also differed after fear extinction [44], with rat IL-PFC H3K9ac significantly higher after delayed extinction than after no or immediate extinction. 

In humans, histone trimethylation differences have been found at various lysine sites in the peripheral blood monocytes of human PTSD subjects [45]. Some of these histone modifications were associated with the differential methylation of certain genes and miRNA expression, especially related to the expression of pro-inflammatory cytokines.

### 3.3. RNA-Associated Silencing

In a mouse model of PTSD, the prefrontal cortex (PFC) miRNA profiles found that traumatic stress alone did not alter long-term miRNA expression compared to controls [46]. However, fluoxetine treatment in traumatized mice significantly reduced several miRNAs, most notably mmu-miR-1971, compared to untreated traumatized mice. This finding would be relevant for future studies of RNA-associated epigenetic mechanisms in PTSD populations with comorbid depressive/anxiety disorders since these comorbidities are frequently treated with selective serotonin reuptake inhibitor (SSRI) antidepressants such as fluoxetine.

### 3.4. Environmental Contributors of Epigenetic Modification

The developing brain undergoes multiple maturational processes including myelin disposition, dendritic pruning, cortical network connectivity and excitability, and GABAergic and glutamatergic receptor functions and interactions. In humans, adverse childhood experiences (ACEs) can include witnessing or experiencing violence, neglect, substance abuse, mental illness, incarceration, or parental separation. Multiple studies have demonstrated a dose-response of ACEs with physical and psychiatric sequelae in later life. ACEs may, therefore, be important contributors to and confounders of studies of epigenetic mechanisms in psychiatric comorbidities. A survey of 9,508 adults found a positive correlation between the number of ACEs and alcohol or drug use disorders, depression, and suicide attempts [47]. These results have been replicated and have also found increased rates of PTSD for each additional ACE [48,49,50]. ACEs are positively correlated not only with childhood PTSD and childhood psychiatric disorders but also with rates of adult exposure to trauma, adult PTSD, and adult psychiatric disorders [51]. In traumatized children, increased methylation in three genes (NMDA glutamate receptor, *GRIN1*; inhibitor of DNA binding 3, *ID3*; and tubulin polymerization promoting protein, *TPPP*) was associated with reduced rates of depression [52]. The importance of comparing data from children with that from adults is that there are developmental changes in glutamatergic receptor–receptor interactions: in early development, NMDA receptors dominate excitatory neurotransmission, whereas the adult brain displays AMPA-NMDA receptor synergism [53]. Decreasing NMDA expression via trauma-induced gene methylation may affect the maturation process of the glutamatergic system. Increased methylation of one CpG island has also been seen in women with higher levels of childhood exposure to domestic violence [54]. 

The intergenerational effect of epigenetic modifications in PTSD subjects is also an essential consideration. Women who were pregnant during the 1995 Rwandan genocide were compared between those in Rwanda versus those out of the country [55]. Compared to out-of-the-country controls and their children, genocide survivors and their children had higher levels of PTSD and depression, lower cortisol levels, and higher DNA methylation of the promoter of *NR3C1* (a glucocorticoid receptor [56]). A similar intergenerational transmission of epigenetic modifications in PTSD has been demonstrated in Holocaust survivors: their elevated *FKBP5* (forkhead binding protein 5) methylation and their children’s decreased *FKBP5* methylation were significantly different from controls [57]. The sample size of this study was a very small and needs replication in a larger cohort of trauma survivors. The mechanisms altering these epigenetic markers are unclear, as is the effect on phenotype; studying a larger cohort would help clarify the significance of these epigenetic markers and account for confounders, including environmental effect and trauma-influenced parenting style.

## 4. Psychiatric Comorbidities

### 4.1. Depression

Dysregulation of the HPA axis occurs in trauma exposure, chronic stress, and depression. In animal models, corticosteroids have an induced DNA demethylation of FK506 binding protein 5 (FKBP5) in neuronal [58] and non-neuronal cells [59]. Chronic steroids also induced a dose-dependent decrease of the DNA methyltransferase enzyme Dnmt1 in mouse hippocampus [59]. FKBP5 is part of the protein complex that co-localizes with the glucocorticoid receptor. It modulates glucocorticoid signaling and has been associated with major depressive disorder (MDD), suicide, and PTSD in the context of high ACEs [30]. FKBP5 regulates glucocorticoid receptor sensitivity, and FKBP5 SNPs have been associated with enhanced HPA axis suppression using the dexamethasone suppression test as well as has predicted the severity of adult PTSD symptoms in the context of childhood trauma [18]. A history of childhood abuse is associated with increased rates of PTSD and depression in the presence of high-risk FKBP5 polymorphisms (rs1360780, rs9296158, rs3800373, and rs9470080), and these are further exacerbated by DNA demethylation [30]. In this complex study, certain risk alleles were found to alter chromatin conformation (and thus gene transcription) relative to protective alleles. Further exploration of the effect of childhood trauma, with its associated epigenetic modifications, was also significantly associated with DNA demethylation of *FKBP5* in risk alleles than in protective alleles. The effects of childhood trauma on DNA demethylation were consistent across European and African-American ethnic groups. The interaction of childhood trauma with adult trauma was more difficult to clarify, given that high ACE scores were associated with higher levels of adulthood traumatic events. The assessment of promoter region methylation of *NR3C1* and *FKBP5* in combat veterans with PTSD undergoing psychotherapy found that, pretreatment, a greater number of methylated sites in the *NR3C1* promoter predicted posttreatment response [60]. The pretreatment methylation of *FKBP5* promoter region did not predict treatment response. However, in responders, the methylation of the FKBP5 promoter region decreased, while it increased in non-responders.

A DNA methylation study examined 473 World Trade Center responders with current MDD or PTSD versus responders who had no lifetime history and found no epigenomic differences reaching significance [61]. However, the differently methylated genes of the two disorders were quite different. PTSD gene methylation was associated with pathways involved in synaptic plasticity, oxytocin signaling, cholinergic synapse, and inflammation, while MDD gene methylation affected phosphatidylinositol signaling and cell cycle pathways. This compares with the methylomic study of urban PTSD subjects with or without MDD +/− generalized anxiety disorder found only 3 genes that were significantly differentially methylated in the presence of comorbidity.

Neurotransmitters transporters have demonstrated differential epigenetic modifications in comorbid depression. The gene *SLC6A3* encodes the dopamine transporter, DAT1. The *SLC6A3* promoter allelic variation has been associated with increased lifetime rates of PTSD (particularly the 9R allele) only in the presence of highly methylated promoter locus at cg13202751 [28]. These individuals also had higher levels of lifetime depression, although not to significance. The increased methylation of the serotonin transporter (*SLC6A4*) promoter has been associated with increased rates of depression symptoms in adolescents with the short-allele of 5-HTTLPR, a polymorphic region within the promoter [62]. The interaction between the HPA-axis and serotonin transporter (SERT or 5-HTT) genotype or 5-HTTLPR genotype in non-human primates and rodents has been shown to be influenced by not only gene variations but also early-life stress and sex [63].

The differential methylation of CpG islands within the brain-derived neurotrophic factor (BDNF) gene has distinguished major depression patients from healthy controls in a Japanese population [64]. BDNF has been implicated in the cognitive and memory deficits of trauma. In an adult rat model of PTSD, exposing rats to predators and social stress reduced growth, altered behavior, and significantly increased dorsal hippocampal *BDNF* promoter methylation with associated transcriptional suppression of *BDNF* [65]. A single-prolonged stress model of PTSD in rats examined the effects of grape powder for its antioxidant properties. It prevented stress-induced corticosterone increases, BDNF decreases, and memory test errors and significantly increased hippocampal and amygdalic H3 acetylation and HDAC5 [66].

### 4.2. Anxiety Disorders

The gene *SKA2* encodes a component of the microtubule-binding complex involved in mitosis. A study of military veterans exposed to at least one lifetime trauma found that methylation at cg13989295 of *SKA2* was associated with higher rates of internalizing disorders (depression, dysthymia, generalized anxiety, phobia, obsessive-compulsive disorder, and panic disorder) and current suicidal ideation and suicidal behaviors [35]. It was not associated with high rates of externalizing disorders (substance use disorders and antisocial personality disorder) or PTSD.

A Swiss study of the fMRI imaging of mothers in controlled stressful parenting situations found that women with higher levels of maternal anxiety had higher levels of methylation at four CpG sites in the *BDNF* promoter [54]. In rats, fear conditioning induced differential histone acetylation in the prefrontal cortex [43]. Fear extinction increased Histone 3 (H3) acetylation in both the infralimbic and prelimbic prefrontal cortex (ILPFC, PLPFC), while H4 acetylation increased only in ILPFC. Neuronal activation (as measured by *c-fos*) followed the same pattern as H3/H4 acetylation.

Rodent models have revealed differential H3 acetylation and DNA methylation in fear studies. In rats that undergo fear extinction after fear training, H3 acetylation was unaffected by immediate extinction training at 10 minutes but significantly increased in the ILPFC after delayed extinction training at 24 hours [44]. The rats also demonstrated region-specific differential neuronal activation: delayed extinction training was associated with a much higher *c-fos* expression in ILPFC but not in PLPFC. In a fear conditioning/extinction paradigm with adult mice, dexamethasone reduced fear behaviors and enhanced extinction learning [67]. Low-dose dexamethasone was associated with FKBP5 mRNA decreases, while high-dose dexamethasone was associated with increased FKBP5 mRNA expression. Additionally, dexamethasone was associated in a dose- and time-dependent manner with increased FKBP5 DNA methylation. In rats, novel environments increase H3 acetylation in the dentate gyrus and benzodiazepines inhibit H3ac [68]. The dentate gyrus is the hippocampal area receiving excitatory neuronal input, especially for novel situations and memory formation, and typically displays pronounced GABAergic inhibitory tone. A partial inverse GABA-A agonist was found to significantly increase H3 acetylation, *c-fos* expression, and anxiety behaviors. These epigenomic changes were prevented by an NMDA receptor antagonist.

Extinguishing fear memories in PTSD subjects has proven difficult, with intense interest in the epigenetic contributions to memory formation and extinction [69]. Fear conditioning in rats caused hippocampal H3K14 (histone H3, lysine 14) acetylation, and HDAC inhibitor administration enhanced fear memory [70]. HDAC inhibitors have also altered rodent stimulant and opiate preferences and aversions, with evidence of modifying memory extinction [71]. 

### 4.3. Psychotic Disorders (Schizophrenia and Bipolar Disorder)

*CACNA1C* encodes a subunit of a calcium channel essential for neuron action potentials and has been implicated in MDD and the psychotic disorders (bipolar disorder and schizophrenia). A Dutch study found that *CACNA1C* CpG-SNP (rs1990322) was significantly associated with PTSD in traumatized police and children and may also interact with the *FKBP5* antagonist *FKBP4* [72]. However, more studies of PTSD with comorbid psychotic disorders need to be undertaken to confirm any association with *CACNA1C* or other epigenetic markers.

### 4.4. Substance Use Disorder

Life-time substance use disorders (SUDs) are estimated at 8–25% of the general population but are much higher in PTSD patients at 21–43% [73]. In one female twin study, 33.3% of women with PTSD and 11.3% of women without PTSD had comorbid alcohol use disorder [74]. Since PTSD is associated with intrusion symptoms that reactivate fear, patients often self-medicate or relapse into prior SUD. There are overlaps in the neural networks that are involved in PTSD and drug-seeking behavior, and research has explored behavioral inhibition circuits and memory. The drugs cocaine and Ayahuasca activate the stress-responsive receptor SIGMAR1 (endoplasmic reticulum protein involved in lipid transport), which reverses memory deficits in animal models, and has been hypothesized to mechanistically contribute to the traumatic memory-retrieval of PTSD patients using Ayahuasca [75]. SIGMAR1 interacts with the histone deacetylases HDAC1/2/3, and this interaction is enhanced by cocaine [76]. Little is known about RNA-associated silencing in PTSD in SUD. The differential expression of lnc-RNA and miRNA has been implicated in altered synaptic plasticity after cocaine administration [69], but this has yet to be linked to PTSD.

### 4.5. Suicide Phenotypes

*SKA2* encodes a protein that regulates mitotic anaphase. *SKA2* methylation in a mostly African-American, urban population was observed to be significantly associated with lifetime suicide attempt, and there was complex interaction between *SKA2* methylation, suicidality, and childhood trauma [36]. *SKA2* methylation in Caucasian veterans post-deployment was associated with higher rates of internalizing symptoms and of suicidal thoughts and behaviors, although it was not significantly associated with PTSD [35]. The decreased methylation of the promoter of glucocorticoid receptor hGR 1_H_ (a differential exon of NR3C1) has previously been associated with completed suicides in victims abused as children [77]. It was re-examined in PTSD subjects, who were found to have hypoactive cortisol responses, possibly secondary to GR hypersensitivity, but suicide status was not explored [78]. 

## 5. Discussion

Epigenetic mechanisms are temporally dynamic, adjusting in the short- and long-term to environmental influences. Gene × environment interactions can influence clinical phenotypes, and these interactions can be further modified by inherited or environmentally-induced epigenetic changes. The low rate of development of PTSD following trauma in most populations contrasted with the much higher rates in populations with childhood trauma, comorbid psychiatric and medical illness, and ancestral trauma emphasizes the complexity of these interactions. Determining the etiology and phenotypy of PTSD requires an understanding of associated environmental influences, neural networks, contributory genes, risk versus protective alleles, and superimposed epigenetic effects. A better understanding of the specific epigenetic contributions of sex and ethnicity in human populations may ultimately help more targeted individual- and population-based prevention and intervention strategies. Clinical psychiatrists can consider additional risk factors during assessments, including enquiring about the transgenerational effects of trauma, the nature and number of ACEs, the importance of demographic profiles, and the effect of psychiatric comorbidities as epigenetic risk factors for PTSD. Collecting this data is also of assistance for the research colleagues, as it provides a more complete repository of information for attempts at correlation between clinical data and genetic/epigenetic profiles. 

Examining human populations to clarify these questions may be complicated by a number of factors. There can be ethnic differences in allele frequency that may affect chromatin confirmation independently of histone or DNA modification. Further complicating genetic analysis is the fact that there are also sex differences in PTSD rates, which may be related to the reliability and/or validity of the diagnosis, possibly related to frequency of symptom reporting [31]. Additionally, different populations face exposure to different types and rates of trauma (e.g., urban versus rural populations, wartime versus peace, and military versus civilian). Military populations are frequently used as a source of PTSD subjects, but it is possible they are not comparable to PTSD cases in the general population, especially when combat veterans have higher rates of physical and traumatic brain injury [79]. There are important differences between military (especially combat personnel) and civilian populations. Although difficult to generalize across all military organizations, some of the differences include significant demographics like age, sex, socioeconomic status, and even race. Within combat units, there can be the protection of intense social cohesion [80], but exposure to life-threatening trauma can be both significantly more severe and frequent than in a typical civilian population, and social cohesion can be lost on return to civilian life. The higher rates of comorbid brain injury increases the risk of PTSD in this population [81]. These differences may be significant in the interpretation of epigenetic markers. Trauma-exposed military veterans have been assessed for DNA methylation, and PTSD hyperarousal was associated with significantly accelerated cellular age as measured by DNA methylation rates [82]. Trauma-associated increased DNA methylation was also associated with a higher probability of all-causes mortality during the 6.5 year follow-up period [82]. Comparing these findings in a civilian population would be helpful to determine the contribution of psychological trauma versus physical effects of combat and injury. On an individual (rather than population) level, an additional confounder may be personality characteristics, such as risk taking or openness to new experiences [74].

Although many clinicians focus on the affective component of PTSD, it is important to consider the perseverative memory component (nightmares and flashbacks). Epigenetic mechanisms are critical in the acquiring and modification of traumatic memories [69]. However, not only do the specific epigenetic modifications in memory formation remain to be understood, many of the rodent fear-model studies need to be replicated in humans. The findings that pre-exposure to stress can alter the nature of fear memories in rodents so that behaviors resemble post-trauma behaviors is important: it aligns with findings in humans that childhood adversity and early trauma are priming factors for future PTSD diagnoses. It is not yet known whether the same epigenetic mechanisms are involved in these different species and whether such mechanisms operate across the vastly different time frames of different species’ youth. Most animal models are able to directly examine brain tissue, while most human studies require peripheral tissues for the indirect assessment of epigenetic modifications occurring in the central nervous system, and though peripheral epigenetic markers frequently reflect central epigenetic changes [83], this is not always the case. All human data reported in this paper was accumulated from peripheral tissues (typically blood or saliva). This further complicates the interpretation of gene associations and epigenetic markers as most peripheral tissue findings have not been replicated in human brain samples. However, there are increasing efforts to establish human postmortem brain collections for PTSD research, such as the US Veteran Administration’s National Posttraumatic Stress Disorder Brain Bank. Another difficulty of limited central nervous tissue availability from humans is that it is difficult to directly study epigenetic changes over the course of human development; hence, a better understanding of animal models of early life stress and trauma will remain an important area of study. Since exposure to stress over the life of an organism is unavoidable, it will be important to understand the mechanisms by which individual and cumulative stressors contribute to or detract from a resilient versus vulnerable phenotype.

## Figures and Tables

**Table 1:** A reported list of protein-coding genes reportedly associated with either an elevated risk of post-traumatic stress disorder (PTSD) or an increased severity of the clinical phenotype of PTSD in human populations. SNP: Single nucleotide polymorphism.

Gene Name (Chromosome, *Homo sapiens*)	Protein Name (Alternative name/s)	Presumptive Protein Role/s	Population Characteristics	Type of Study	Association with PTSD
*ADCY8* Ch 8q24.22	Adenylate cyclase 8 (ADCY8)	Catalyses cAMP formation from ATP	484 White, non-Hispanic, trauma-exposed military veterans and their civilian partners	SNP genotype	SNP association: rs263232 in *ADCY8* associated (not to genome-wide significance) with dissociative symptoms of PTSD [25].
*ADCYAP1R1* Ch 7p14.3	ADCYAP receptor type 1 (PAC1)	May regulate release of adrenocorticotropin, LH, GH, PRL, epinephrine	798 subjects, various races, trauma-exposed civilians (36.9% male)	SNP genotype	SNP association: rs2267735 CC genotype predicts PTSD diagnosis and symptom severity in female humans [26].
*ANKRD55* Ch 5q11.2	Ankyrin repeat domain 55 (ANKRD55)	Role in juvenile arthritis	10,834 subjects, various races, military personnel (80.7% male)	Genome-wide association study	SNP association: rs159572 in African-Americans was associated with PTSD diagnosis [15].
*BRSK1* Ch 19q13.42	BR Serine/threonine kinase 1 (BRSK1)	Neuron polarization, centrosome duplication	211 subjects, 96 male Australian combat veterans, 115 male general population subjects	Genome-wide methylation analysis	Differential methylation: DNA hypomethylation of CpGs spanning the gene was associated with greater PTSD symptom severity [17].
*CNR1* Ch 6q15	Cannabinoid receptor 1	G-protein coupled receptor, inhibits adenylate cyclase	187 children with ADHD (69.5% male), 374 parents (50% male), various races	SNP genotype	SNP association: rs1049353 allele A showed significant association with PTSD diagnosis in Caucasian parents [27].
*DAT1/SLC6A3* Ch 5p15.33	Dopamine active transporter (DAT, SLC6A3)	Dopamine transport	1547 adults (362 trauma exposed), various races	SNP genotype, DNA methylation microarray	SNP association: *DAT1* 39UTR VNTR 9R allele doubles lifetime risk of PTSD but only in conjunction with high methylation in the DAT1 promoter locus [28].
*DDX60L* Ch 4q32.3	DExD/H-Box 60 Like (DEAD Box protein 60-like)	Helicase, mediates ATP binding/hydrolysis, nucleic acid binding, RNA unwinding	1929 military veterans, 383 mixed population of civilians and veterans, various races	Genome-wide association study	SNP association: On meta-analysis, intronic rs10002308 significantly associated with increased risk for PTSD diagnosis [16].
*DOCK2* Ch 5q35.1	Dedicator of cytokinesis 2 (DOCK2)	Remodels actin cytoskeleton in hematopoietic cells	211 subjects, 96 male Australian combat veterans, 115 male general population subjects	Genome-wide methylation analysis	Differential methylation: DNA hypomethylation of CpGs spanning the gene was associated with greater PTSD symptom severity [17].
*DPP6* Ch 7q36.2	Dipeptidyl-aminopeptidase-like protein 6 (DPP6)	Bind voltage gated K+ channels	484 White, non-Hispanic, trauma-exposed military veterans and their civilian partners	SNP genotype	SNP association: rs71534169 in *DPP6* associated (not to genome-wide significance) with dissociative symptoms of PTSD [25].
*DRD2* Ch 11q23.2	Dopamine receptor D_2_ (DRD2, D2R)	D2 subtype of dopamine neurotransmitter receptor, inhibits adenylyl cyclase	56 combat veterans, mean age 43.6 years [13] 52 European-American combat veterans, 87 European-American controls [29]	SNP genotype [13] Association study [29]	Allelic association: DRD2 A1 allele significantly associated with PTSD [13]. Result not replicated [29].
*DSCAM* Ch 21q22.2	Down Syndrome Cell Adhesion Molecule (DSCAM)	Cell adhesion molecule, neuronal self-avoidance	1929 military veterans, 383 mixed population of civilians and veterans, various races	Genome-wide association study	SNP association: In African-Americans, rs77290333 increased risk for PTSD diagnosis [16].
*FKBP5* Ch 6p21.31	Forkhead binding protein 5 (FKBP5, FK506 binding protein 5, Immunophilin)	Immunoregulation, protein folding, protein trafficking	900 trauma-exposed adults, 42.7% male, 95.2% black [18] 30 trauma-exposed adults, mean age 41.46, 27 African-American; 46 controls, mean age 40.97, 45 African-American [30]	SNP genotype [18] SNP genotype [30]	SNP association: FKBP5 SNPs (rs9296158, rs3800373, rs1360780, rs9470080) showed significant gene × environment (severity of childhood sexual abuse) interaction predicting severity of adulthood PTSD symptoms and enhanced glucocorticoid receptor sensitivity [18]. Allelic variation interacts with ACE-induced demethylation to affect gene transcription [30].
*KLHL1* Ch 13q21.33	Kelch-like family member 1 (KLHL1)	Possible actin binding	20,730 subjects from 11 combined studies	Genome-wide association study	SNP association: In African-Americans, rs139558732 increased risk for PTSD diagnosis [31].
*LCN8* Ch 9q34.3	Lipocalin 8 (LCN8, lipocalin 5, LCN5)	Binds and transports hydrophobic ligands	211 subjects, 96 male Australian combat veterans, 115 male general population subjects	Genome-wide methylation analysis	Differential methylation: DNA hypomethylation of CpGs spanning the gene was associated with greater PTSD symptom severity [17].
*NGF* Ch 1p13.2	Nerve growth factor (NGF)	Nerve growth stimulation and regulation	211 subjects, 96 male Australian combat veterans, 115 male general population subjects	Genome-wide methylation analysis	Differential methylation: DNA hypomethylation of CpGs spanning the gene was associated with greater PTSD symptom severity [17].
*NR3C1* Ch 5q31.3	Glucocorticoid receptor (GR, GCR, NR3C1)	Transcription factor, transcription regulator	118 combat veterans diagnosed with PTSD mean age 55.7, 42 combat exposed non-PTSD controls, mean age 61.2	Glucocorticoid receptor polymorphism analysis	Allelic association: *Bcl*I GG genotype associated with low basal cortisol in PTSD subjects [11].
*OR11L1* Ch 1q44	Olfactory receptor family 11 subfamily L member 1 (OR11L1)	Receptor for odorants to trigger smell perception	619 Mexican American adults; unspecified number of American-Indian adults	Genome-wide association study	Allelic association: Six variants in *OR11L1* associated with risk of PTSD in Mexican-Americans [26].
*OR2L13* Ch 1q44	Olfactory receptor family 2 subfamily L member 13 (OR2L13)	Receptor for odorants to trigger smell perception	619 Mexican-American adults; unspecified number of American-Indian adults	Genome-wide association study	SNP association: rs151319968 associated with PTSD in Native American sample [32].
*PRKG1* Ch 10q11.23	Protein kinase CGMP- dependent type 1 (PRKG1B)	Mediates nitric oxide/cGMP signaling, modulates cell growth, regulates neuron function	1929 military veterans, 383 mixed population of civilians and veterans, various races	Genome-wide association study	SNP association: On meta-analysis, intronic rs10762479 was significantly associated with increased risk for PTSD diagnosis [16].
*PRTFDC1* Ch 10p12.1	Phosphoribosyl transferase domain containing 1 (PRTFDC1, HHGP)	Protein homodimerization, magnesium ions	3494 male US Marines, 85.5% white, mean age 23.1	Genome-wide association study	SNP association: rs6482463 in *PRTFDC1* associated with PTSD across ancestry groups [33].
*RORA* Ch 15q22.2	RAR-related orphan receptor alpha (RORα, NR1F1)	Nuclear hormone receptor	852 military veterans and their partners; 435 trauma-exposed subset, 59.7% male	Genome-wide association study	SNP association: rs8042149 associated with lifetime diagnosis of PTSD [34].
*SDC2* Ch 8q22.1	Syndecan 2 (SYND2, HSPG)	Cell binding, cell signaling, cytoskeletal organization	1929 military veterans, 383 mixed population of civilians and veterans, various races	Genome-wide association study	SNP association: In Caucasians, rs2437772 increased risk for PTSD diagnosis [16].
*SKA2* Ch17q22	Spindle And Kinetochore Associated Complex Subunit 2 (FAM33A)	Chromosome segregation during mitosis	466 White, non-Hispanic, trauma-exposed military veterans and their civilian partners (65% male) [35] 421 subjects from the Grady Trauma Project and 326 subjects from Johns Hopkins Center Prevention Research Study [36]	Genotyping and methylation analysis [35] Genotyping and methylation analysis [36]	Differential methylation: Methylation at CpG locus cg13989295 associated with higher levels of internalizing disorders [35]. Differential methylation: SKA2 methylation predicted lifetime suicide attempt and cortisol suppression and interacted with ACE to predict PTSD [36].
*SLC6A4* Ch 17q11.2	Serotonin transporter (5-HTT, SERT, SLC6A4)	Serotonin transport from synapse into presynaptic neurons	589 adults from Florida Hurricane Study, 36.5% men, 90% white [14] 45 PTSD adults in 8 week cognitive behavior therapy program [12]	Genotyping [14] 5-HTTLPR genotype [12]	Allelic association: S allele linked to higher PTSD risk in context of poor social support [14]. Allelic association: S allele had greater PTSD symptom severity and poorer response to CBT [12].
*TBC1D2* Ch 9q22.33	TBC1 domain family member 2 (TBC1D2)	Vesicle transport, cell–cell adhesion	1929 military veterans, 383 mixed population of civilians and veterans, various races	Genome-wide association study	SNP association: In Caucasians, rs7866350 increased risk for PTSD diagnosis [16].
*TLL1* Ch 4q32.3	Tolloid-like protein 1 (TLL1)	Metalloprotease that processes procollagen C-propeptides	9340 subjects; 1040 with PTSD, 5947 controls, various races	Genome-wide association study	SNP association: rs6812849 and rs7691872 in the first intron of *TLL1* in European Americans were associated (not to genome-wide significance) with lifetime PTSD diagnosis [37].
*UNC13C* Ch 15q21.3	Unc-13 Homolog C (UNC13C)	Vesicle maturation, exocytosis	1929 military veterans, 383 mixed population of civilians and veterans, various races	Genome-wide association study	SNP association: In African-Americans, rs73419609 increased risk for PTSD diagnosis [16].
*ZNF626* Ch 19p12	Zinc finger protein 626 (ZNF626)	Possibly transcription regulation	10,834 various races, military personnel (80.7% male)	Genome-wide association study	SNP association: rs11085374 in European Americans was significantly associated with PTSD diagnosis [15].
